# The modulatory role of cannabis use in subconcussive neural injury

**DOI:** 10.1016/j.isci.2023.106948

**Published:** 2023-05-25

**Authors:** Rachel M. Kalbfell, Devin J. Rettke, Ken Mackie, Keisuke Ejima, Jaroslaw Harezlak, Isabella L. Alexander, Jim Wager-Miller, Blair D. Johnson, Sharlene D. Newman, Keisuke Kawata

**Affiliations:** 1 Department of Kinesiology, Indiana University School of Public Health-Bloomington, Bloomington, IN, USA; 2 Bioethics Research Center, Washington University School of Medicine in St. Louis, St. Louis, MO, USA; 3 Department of Psychological and Brain Sciences and Gill Center for Molecular Bioscience, The College of Arts and Sciences, Indiana University, Bloomington, IN, USA; 4 Program in Neuroscience, The College of Arts and Sciences, Indiana University, Bloomington, IN, USA; 5 Lee Kong Chian School of Medicine, Nanyang Technological University, Singapore, Singapore; 6 Department of Epidemiology and Biostatistics, Indiana University School of Public Health-Bloomington, Bloomington, IN, USA; 7 Alabama Life Research Institute, University of Alabama, Tuscaloosa, AL, USA

**Keywords:** Clinical medicine, Clinical neuroscience, Plants

## Abstract

Cannabis use has become popular among athletes, many of whom are exposed to repetitive subconcussive head impacts. We aimed to test whether chronic cannabis use would be neuroprotective or exacerbating against acute subconcussive head impacts. This trial included 43 adult soccer players (Cannabis group using cannabis at least once a week for the past 6 months, n = 24; non-cannabis control group, n = 19). Twenty soccer headings, induced by our controlled heading model, significantly impaired ocular-motor function, but the degrees of impairments were less in the cannabis group compared to controls. The control group significantly increased its serum S100B level after heading, whereas no change was observed in the cannabis group. There was no group difference in serum neurofilament light levels at any time point. Our data suggest that chronic cannabis use may be associated with an enhancement of oculomotor functional resiliency and suppression of the neuroinflammatory response following 20 soccer headings.

## Highlights

• Cannabis users showed oculomotor resiliency to acute headings
• Cannabis use may attenuate post-heading astrocyte activation
• Serum neurofilament light increased only in non-cannabis users

## Graphical abstract

**Figure:**
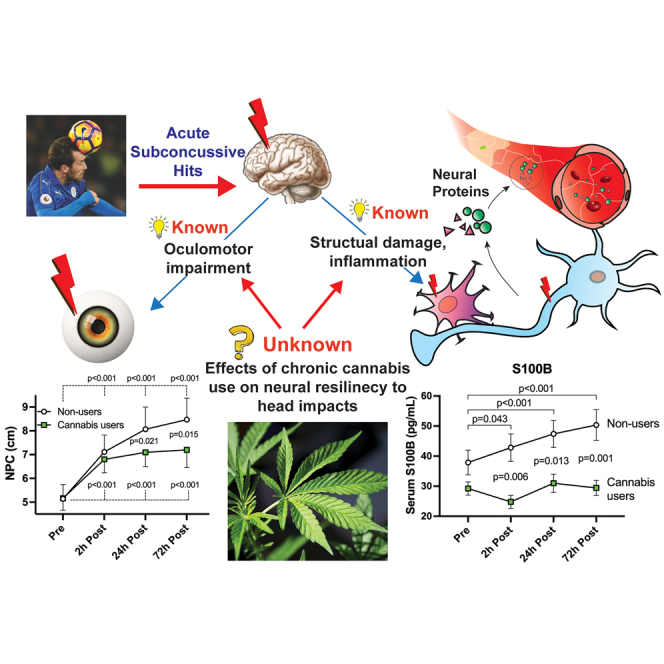

## Introduction

The legalization of medical and recreational cannabis use is sweeping across the United States. In recent years, cannabis use has gained significant popularity among adult athletes due to many anecdotal and some research reports indicating cannabis’s positive effects on chronic pain,^1^ mental stress,^2^ and neurodegenerative diseases.^3^ Furthermore, the National Football League in 2020 increased the allowable level of tetrahydrocannabinol (THC), the psychoactive compound in cannabis, from 35 to 150 ng/mL to allow players to use cannabis to cope with their mental and physical pain.^4^ The exact same policy on the THC threshold has been adopted by the National Collegiate Athletic Association (NCAA) in 2021.^5^ Other professional organizations (e.g., NBA, MLB, and NHL) have relaxed their stance on cannabis use among players too. Meanwhile, athletes in contact sports can sustain hundreds of subconcussive head impacts annually.^6^ These impacts are defined as hits to the head or body without eliciting overt signs and symptoms of a concussion, whereas a concussion can induce a range of clinical signs and symptoms, as well as short- and long-term impairments in neurological function.^7^ The increasing use of cannabis in athletes exposed to head impacts raises the question: “Does habitual cannabis use exacerbate the negative neural effects of head impacts, or is it neuroprotective?”

The question of whether cannabis use has a positive or negative impact on the brain is incredibly difficult to answer because of the varied age of onset of use, products with highly variable levels of THC and other cannabinoids, and heterogeneous exposure (dose, duration, and route). As a result, evidence from meta-analysis and systematic reviews diverges, suggesting both pros and cons. For example, a retrospective cohort study of 446 patients with severe traumatic brain injury (TBI) found that, after controlling for age, injury mechanism, sex, and alcohol, the mortality rate was significantly lower for the THC-positive group (2.4%, hazard ratio of 0.224) than for the THC-negative group (11.5%).^8^ A positive THC test in patients with TBI was also associated with shorter ICU stay and ventilator needs.^9^ The potential positive effects of cannabis use were also noted in post-concussive symptoms, such that patients with concussions who recreationally used cannabis showed a lower symptom burden at week 3 and 4 of concussion recovery.^2^ Three to six months of medical cannabis treatment in patients with at least one medical or psychiatric indication (e.g., anxiety, sleep, mood, and chronic pain) showed significant improvements in white matter coherence, and this improvement was more driven by cannabidiol than THC.^10^ A Phase II clinical trial in patients with severe TBI using a synthetic, non-psychotropic cannabinoid has led to an acute reduction in intracranial pressure and cerebral perfusion pressure and improvement in Glasgow Outcome Scale scores.^11^ However, the follow-up Phase III clinical trial showed no differences in neurologic outcomes between placebo and intervention groups at 6-month follow-up.^12^ Other lines of research also suggest that cannabis use is associated with cognitive impairments, altered neuronal connectivity,^13^^,^^14^ reduced white matter coherence, and reduced axonal microstructural integrities.^15^^,^^16^^,^^17^

The biological effects of cannabis on neurotrauma have been explored in *in vitro* and *in vivo* rodent TBI models.^18^^,^^19^ For example, Zhang et al.^20^ applied a mild cortical impact for 3 consecutive days in mice, which significantly reduced glutamate receptors and ionic channels (e.g., GluA1, GluA2, and GluN2A) and increased proinflammatory cytokines (e.g., IL-6 and TNF-α). These TBI effects were reversed to levels similar to a sham cohort by increasing endogenous cannabinoid levels with a monoacylglycerol-lipase (MAGL) inhibitor, which mimics the effects of cannabis administration. Indirectly activating cannabinoid signaling by MAGL and alpha/beta-hydrolase domain-containing-6 inhibitors resulted in the protection of long-term potentiation at hippocampal synapses,^20^ inhibition of glial activation,^21^ and preservation of blood-brain barrier integrity.^22^ Collectively, these data support the hypothesis that cannabis is a potentially effective countermeasure for TBI; however, it has become clear that the clinical evidence is at its infancy stage, warranting a well-controlled mechanistic study.

To begin addressing our research question, we designed and conducted an intervention trial comparing chronic cannabis users and non-cannabis users and employed our innovative soccer-heading model to isolate subconcussive effects.^23^^,^^24^^,^^25^^,^^26^^,^^27^^,^^28^^,^^29^^,^^30^ This model can induce standardized stimuli across human subjects, allowing us to mechanistically study the interactive effects of cannabis and subconcussive head impacts, while eliminating extraneous influences that are inherent in field studies, such as bodily hits, fatigue, strenuous exercise, and perspiration/hydration.^23^ We selected near-point of convergence (NPC), which measures the closest point of focus before diplopia occurs,^24^^,^^26^ as our clinical outcome measure, whereas serum levels of S100B and neurofilament light (NfL) were assessed to gauge astrocyte activation and axonal injury,^31^^,^^32^ respectively. These measures were chosen because of their sensitivity in detecting subconcussive injury,^24^^,^^25^^,^^26^^,^^27^^,^^28^^,^^29^^,^^30^ as compared to other measures, such as balance and cognition. Since studies demonstrate cannabis’s potent beneficial effects in brain injury,^2^^,^^33^ we hypothesized that chronic cannabis use would mitigate elevations of NPC, S100B, and NfL levels after 20 acute headings, whereas non-cannabis users would show significant elevations in NPC, S100B, and NfL.

## Results

### Demographic and head impact kinematics

**Figure 1:**
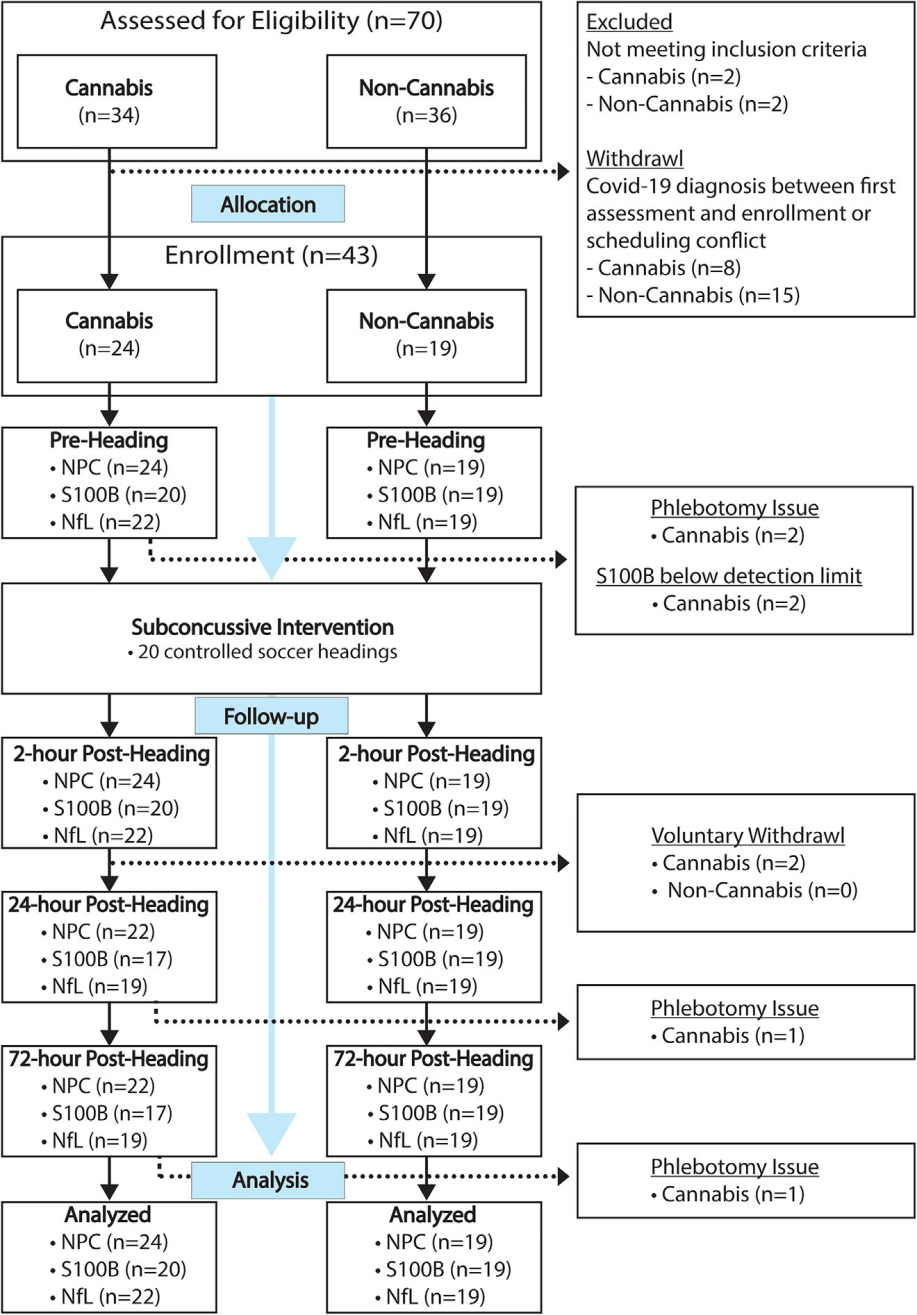
Study flow chart

Seventy individuals were evaluated for eligibility. Twenty-three individuals (n = 8 cannabis, n = 15 non-cannabis) were excluded from the study due to COVID-19 diagnosis occurring between the day of eligibility screening and the study data collection (pre-heading baseline), or scheduling conflict. Two subjects voluntarily withdrew after the 2-h post-heading time point, but their data were included in the analysis since the mixed-effects regression model can accommodate missing values in the analysis. As a result, data from 43 participants (n = 24 cannabis, n = 19 non-cannabis) were available for analysis (Figure 1). There were no significant group differences in demographic factors and impact kinematics, except for CUDIT-R scores as expected (Table 1).

**Table 1:** Demographic characteristics

Variables	Cannabis	Non-Cannabis	P-value
N	24	19	
Sex	17M 7F	10M 9F	0.186
Age, y	20.42 ± 1.88	20.47 ± 1.74	0.946
BMI, kg/m^2^	23.73 ± 4.15	23.79 ± 2.84	0.973
No. of previous concussion			0.314
0, n (%)	13 (54.2)	15 (78.9)	
1, n (%)	8 (33.3)	3 (15.8)	
2, n (%)	2 (8.33)	0 (0.00)	
3, n (%)	1 (4.17)	1 (5.3)	
Soccer heading experience, y	10.92 ± 3.31	9.89 ± 3.51	0.325
Race, n (%)			0.668
White	22 (91.7)	18 (94.7)	
Black/African American	1 (4.17)	0 (0)	
Asian	1 (4.17)	1 (5.3)	
Ethnicity, n (%)			0.723
Non-Latino/Hispanic	19 (79.2)	14 (73.7)	
Latino/Hispanic	5 (20.8)	5 (26.3)	
Corrective Lens (%)	11 (45.8)	6 (31.6)	0.525
AUDIT	6.17 ± 3.43	4.47 ± 3.32	0.111
CUDIT-R	11.75 ± 5.84	0.00 ± 0.00	<0.001
Heading impact kinematics per heading			
Peak linear acceleration, g, mean (SD)	13.56 ± 1.69	14.10 ± 1.94	0.334
Peak rotational acceleration, rad/s^2^, mean (SD)	723.44 ± 216.97	834.4 ± 220.5	0.110
Frequency of cannabis use, n (%)			
At least once a week	4 (16.7)	–	
2–3 times per week	9 (37.5)	–	
4 or more times per week	11 (45.8)	–	
Mode of cannabis use, n (%)			
Vaping/Smoking	10 (41.7)	–	
Edibles	1 (4.2)	–	
Vaping/Smoking/Edibles	10 (41.7)	–	
Vaping/Smoking/Oil	1 (4.2)	–	
Vaping/Smoking/Edibles/Oil	2 (8.3)	–	

BMI, body mass index (calculated as weight in kilograms divided by height in meters squared); AUDIT, Alcohol Use Disorders Identification Test; CUDIT-R, Cannabis Use Disorders Identification Test-Revised.

### Group difference in the clinical marker – NPC

**Figure 2:**
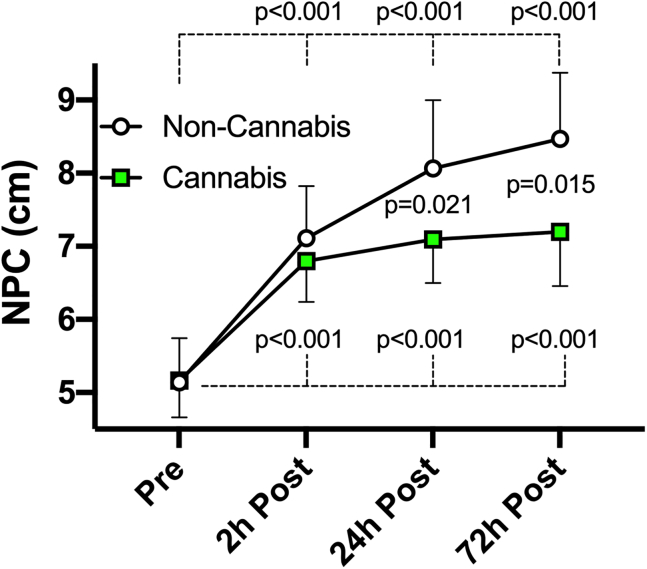
Head impact effects on oculomotor function Twenty soccer headings significantly worsened NPC in both groups. Group-by-time interactions appeared at 24- and 72-h post-heading, as the cannabis group showed significantly smaller degrees of NPC worsening compared to the non-cannabis group. Data are presented as mean ± SEM.

Twenty bouts of soccer headings significantly elevated (worsened) NPC at all post-heading time points in both groups compared to their baseline levels (see Table 2 for within-group changes). The effect of cannabis was illustrated by significant group-by-time interactions detected at 24- and 72-h post-heading, in a way that the cannabis group showed fewer degrees of NPC impairment than the non-cannabis group [24-h, −1.23 cm (−2.27, −0.2), p = 0.017; 72-h, −1.32 cm (−2.36, −0.27), p = 0.015; Figure 2].

**Table 2:** Within-group changes from pre-heading baseline in clinical and biochemical outcomes

Variables	Group	2 h Post (compared to baseline)	24 h Post (compared to baseline)	72 h Post (compared to baseline)
NPC (Unit in cm)	Cannabis user	1.66 (0.99, 2.33) p < 0.001	1.88 (1.19, 2.57) p < 0.001	2.04 (1.34, 2.75) p < 0.001
	Non-user	2.08 (1.3, 2.87) p < 0.001	3.11 (2.34, 3.88) p < 0.001	3.36 (2.59, 4.13) p < 0.001
S100B (Unit in pg/mL)	Cannabis user	−4.44 (−9.06, 0.17) p = 0.062	0.71 (−4.18, 5.6) p = 0.777	0.78 (−4.11, 5.67) p = 0.755
	Non-user	4.95 (0.22, 9.68) p = 0.043	9.52 (4.79, 14.25) p < 0.001	12.49 (7.76, 17.22) p < 0.001
NfL (Unit in pg/mL)	Cannabis user	−0.25 (−0.61, 0.1) p = 0.164	−0.09 (−0.46, 0.29) p = 0.656	0 (−0.37, 0.38) p = 0.992
	Non-user	−0.21 (−0.6, 0.17) p = 0.276	0.35 (−0.04, 0.73) p = 0.079	0.42 (0.04, 0.8) p = 0.034

Values expressed as difference from pre-heading baseline, followed by 95% confidence interval (low, high) and p value. NPC, near point of convergence. NfL, neurofilament light.

### Group differences in the biochemical markers – S100B and NfL

**Figure 3:**
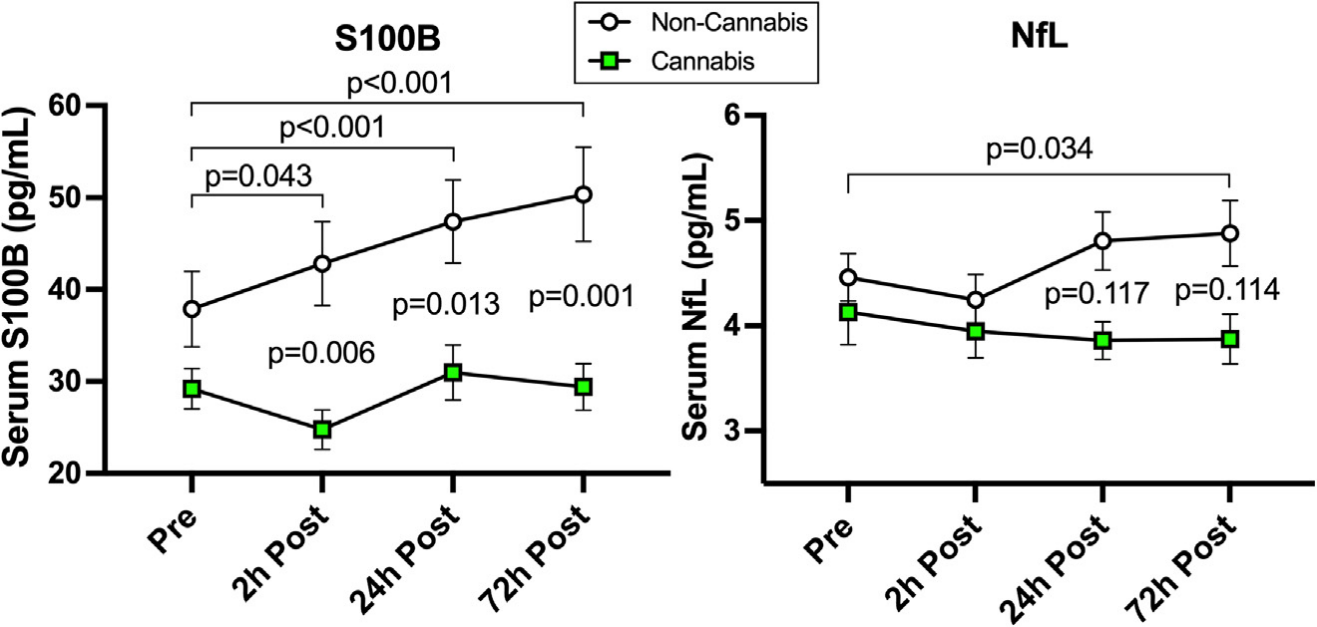
Head impact effects on blood biomarkers Group difference in blood biomarker response to 20 soccer headings. The non-cannabis group showed significant increases in S100B levels at each time point following 20 soccer headings, whereas no changes observed in the cannabis group, which resulted in significant group-by-time interactions at 2-, 24-, and 72-h post-heading. Although the non-cannabis group showed mild elevation in serum NfL levels at 72-h post-heading, there was no between-group difference at any time. Data are presented as mean ± SEM.

The effect of cannabis use was also supported by the serum level of S100B but not NfL (Figure 3). Specifically, 20 soccer headings led to a significant increase in S100B levels in the non-cannabis group at all post-heading time points compared to pre-heading, and the highest expression was observed at 72-h post-heading (see Table 2 for within-group changes). Conversely, S100B levels in the cannabis group remained consistent throughout the study time points. The cannabis group consistently showed lower levels of S100B at post-heading time points, as illustrated by the group-by-time interactions at all post-heading time points [2-h, −9.4 pg/mL (−16, −2.79), p = 0.006; 24-h, −8.81 pg/mL (−15.61, −2.01), p = 0.013; 72-h, −11.71 pg/mL (−18.51, −4.91), p < 0.001].

In contrary to S100B, serum levels of NfL showed no significant within- or between-group differences, except for a within-group elevation observed at 72-h post-heading in the non-cannabis group compared to pre-heading [0.42 pg/mL (0.04, 0.8), p = 0.034; Table 2 and Figure 3].

### The classification abilities of clinical and biochemical markers

**Figure 4:**
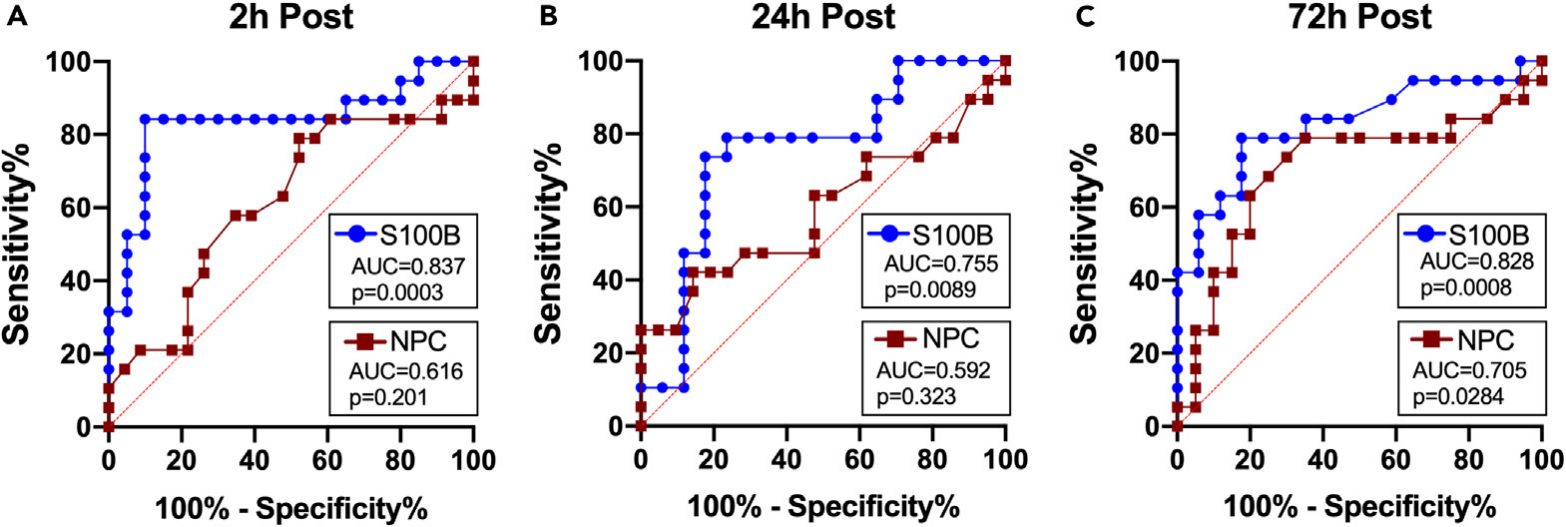
Classification accuracy ROC curves of S100B and NPC at each post-heading time point. Very good classification (diagnostic) capabilities were identified in serum S100B at (A) 2-, (B) 24-, and (C) 72-h post-heading. Conversely, NPC showed only fair/good ability to distinguish between the cannabis-user and non-user groups at 72-h post-heading.

A post hoc ROC analysis was conducted on NPC and S100B that showed significant group-by-time interactions. The analysis revealed that serum levels of S100B had very good accuracy for distinguishing between the cannabis and non-cannabis groups at all post-heading time points [2-h, AUC = 0.837 (0.698, 0.976), p = 0.0003; 24-h, AUC = 0.755 (0.588, 0.923), p = 0.0089; 72-h, AUC = 0.828 (0.691, 0.966), p = 0.0008]. On the other hand, the classification capability of NPC was fair at 72-h post-heading [AUC = 0.705 (0.530, 0.881), p = 0.0284] but no other time points [2-h, AUC = 0.616 (0.439, 0.792), p = 0.202; 24-h, AUC = 0.592 (0.408, 0.775), p = 0.323; Figure 4].

## Discussion

Cannabis use has faced divergent scientific views. Cannabis has shown its potential in ameliorating physical, neurological, and psychiatric symptoms,^2^^,^^34^^,^^35^^,^^36^ whereas other lines of research raised concerns about its safety related to cannabis use disorder and development of psychosis, as well as its impact on the developing brain.^37^^,^^38^^,^^39^ To delineate the interaction between cannabis use and subconcussive neural injury, the current study used multimodal clinical and biochemical markers while isolating the effects of head impact by controlling for the head impact frequency and intensity using the soccer heading model.^23^ This eliminates the concerns surrounding S100B data in response to exercise and body injury that is inherent to field studies. Our data showed that cannabis use blunted oculomotor impairments and prevented astrocyte over-activation. Specifically, NPC continued to increase at least up to 72 h post 20 headings in the non-cannabis group while such NPC elevation plateaued after 24 h in the cannabis group. The group difference was more conspicuous in serum S100B levels, where the non-cannabis group linearly increased S100B levels throughout the study time points. However, the cannabis group showed almost no change in the S100B level, which resulted in a very good classification accuracy for groups at all post-heading time points. Conversely, although the non-cannabis group showed a modest within-group elevation in serum NfL levels at 72-h post-heading, there was no group difference at any time point. Taken together, the current study presents the first empirical evidence in humans that chronic cannabis use may be associated with an enhancement of oculomotor functional resiliency and suppression of neuroinflammatory responses.

NPC and S100B reflect different aspects of brain health, but their time-course data suggest a hypothesis that cannabis use may bolster anti-inflammatory properties in the brain to counteract secondary injury, which can lead to expedited recovery and milder functional deficits. This hypothesis has been supported by both clinical and preclinical studies. The existing clinical studies, unlike our approach, are largely observational, yet several studies showed cannabis’ protective effects against catastrophic brain injury. For example, Nguyen et al.^8^ conducted a retrospective review of patients with TBI in a Level 1 Trauma Center and discovered that patients with TBI with positive THC screening had significantly lower mortality rate (2.4%) compared to patients with TBI with negative THC (11.5%). Subsequent studies by other groups corroborated the findings that patients with trauma with a positive THC urine toxicology test had shorter lengths of stay in the ICU,^9^ as well as lower mortality rate (4.3% vs. 7.6%) compared to non-THC counterparts.^9^^,^^40^ In a different line of research, a Phase III clinical trial delivered a single 150 mg dose of dexanabinol, a synthetic cannabinoid, to patients with severe TBI and found that cannabis treatment did not lead to improvement in any functional (Glasgow Outcome Scale) and physiological outcomes (e.g., intracranial pressure) at 6 months^12^ This discrepancy may be because dexanabinol has N-Nitrosodimethylamine antagonist effects while such effects in recreational cannabis products may be smaller than dexanabinol. It is important to note that all participants in our study have recreationally used cannabis, which is distinctly different from synthetic cannabinoids or derivatives used in more regulated clinical trials and pre-clinical studies. Therefore, caution is required to compare findings across studies using different compounds. In an observational study, Lawrence et al.^2^ tested an interaction between cannabis use and recovery duration in patients with concussions. In a pool of 307 concussed patients, 75 patients (24.4%) reported regular cannabis use prior to the concussive injury and 43 patients continued to use cannabis products during the acute phase of recovery. Their analyses revealed no significant interaction between acute cannabis use and the speed of cognitive and physical recoveries determined by physicians’ evaluation. Conversely, among concussed patients with lingering symptoms, cannabis users showed lower overall symptom scores at 3- and 4-week post-concussion compared to non-cannabis users.

The potential mechanism underlying the effects of cannabis was introduced in several preclinical studies. For example, one study induced inflammatory brain damage via lipopolysaccharide injection in rats; however, a low dose of THC 48 h prior to the injury led to suppression of cyclooxygenase-2, which indicates an anti-inflammatory response, and a better score in an object recognition test compared to injured rats without THC.^41^ Contrarily, another line of research supports the use of cannabis after TBI. Bhatt et al.^42^ induced repetitive lateral cortical impacts with an interval of 3–4 days in rats, followed by treatment with a daily injection of THC for 12 days. These rats with THC treatment showed significant elevations in gene expressions that are known to mitigate excitotoxicity (e.g., *Comt*) and repair damaged vasculature (e.g., *Vegf-2R*). This molecular response was translated into behavioral outcomes, where THC treatment reduced anxiety and depression-like behaviors and improved short-term working memory. Additional evidence suggests that compounds influencing the endocannabinoid cascade (e.g., GP1a, HU-910, and HU-914) after TBI have led to (i) inhibition of neuroinflammation by suppressing tumor necrosis factor alpha production^43^ and converting of macrophage polarization from a pro-to an anti-inflammatory state,^21^^,^^44^ (ii) improvement of neurobehavioral function, and iii) increased synaptogenesis.^43^ However, it remains unclear as to why the Phase III clinical trial failed^12^ despite ample preclinical evidence supporting the use of cannabis in the TBI population. It may be because it requires habituation and multiple doses of cannabis treatment, instead of a single dose given in the clinical trial.^12^ Our rigorously controlled data in subconcussive neural injury in humans provide a meaningful contribution to the ongoing discussion of whether cannabis use is safe, beneficial, or unsafe in the TBI population.

One unexpected finding was that serum NfL levels in the non-cannabis (control) group did not show as robust of an elevation as they did in the previous soccer heading studies^30^^,^^45^ as well as field-based studies in football^46^^,^^47^ and boxing.^48^ A possible reason may be that our NfL was assessed in the semi-automated benchtop SR-X device as opposed to fully automated SIMOA HD-1 analyzers used in other studies. Another reason may be due to the expression difference between serum (the current study) and plasma (the previous heading studies^30^^,^^45^). Although marginal, a significant elevation was observed in the non-cannabis group at 72 h, pointing to the possibility of needing a greater sample size for NfL experiments.

### Clinical implications

The effects of repetitive head impacts have gained increased attention primarily due to the early onset of neurodegenerative cases found in boxers, American football, and soccer players. Notably, clinical studies have found male professional soccer players to have an increased risk of developing neurodegenerative diseases by 3-fold, especially Alzheimer disease, compared to the general population,^49^ with the risk increasing along with the number of years played.^50^ The last decade of research has shed light on the potential link between repetitive subconcussive head impacts in American football and progressive tauopathy, chronic traumatic encephalopathy.^51^ Given that many contact sport athletes are exposed to hundreds of head impacts in a season,^6^ they endure physical pain alongside mental stress burden on a daily basis, which is one of the reasons why cannabis use has spiked in athletes. Just as the NFL’s softened policy related to the THC threshold, the exact same policy has been adopted by the NCAA in 2021.^5^ These policy changes, epidemiological trends, and clinical data emphasize the need and significance of a well-controlled mechanistic human study, such as the current study. Our data offer insights into the role of cannabis against subconcussive neural injury, which sets the stage for large-scale field-based studies.

### Limitations of the study

There are various limitations to acknowledge in this study. First, the study design observes the participants for 72 h post-heading; therefore, we are unable to evaluate how long the elevation of NPC and S100B lasted before returning to baseline for non-cannabis users. Another limitation of the study is the lack of ecological validity of our heading intervention, in which participants performed 20 soccer headings with 30-s intervals in a 10-min time span. However, this study design and methodology is one of the purest ways to isolate the effects of head impacts to mechanistically study the interaction effects between cannabis use and head impacts while limiting potential confounding factors. A future study in a field setting is warranted to test the translatability of our findings. While participants were told to refrain from using other types of recreational drugs, no other drug toxicology test was performed. Therefore, more rigorous screening would help form homogeneous samples in the study groups. Lastly, it is possible to conduct a randomized controlled trial that uses a standardized cannabis product and dosages. Such a study will require several layers of regulatory board approvals and substantial institutional support since cannabis and cannabinoid are scheduled substances under the Controlled Substance Act. Instead, we chose a mechanistic trial design with a case-control comparison to test our hypothesis. This approach coupled with a small set of questionnaires related to cannabis use introduced additional limitations, such that more detailed information regarding specific cannabinoids and their constituents, potency, frequency, and dose of cannabis use should be incorporated in future studies. These factors may mediate the study findings. Despite this limitation, strong group differences observed in our repeated measures design support the validity of our findings. It may be worth considering future clinical approaches focusing on randomized control trials.

### Conclusion

Cannabis use and subconcussive head impacts among young adults are prevalent medical concerns, yet in recent years, these issues began occurring concomitantly. This current study has rigorously isolated subconcussive effects and assessed for the potential interaction effects between cannabis use and head impacts on the brain. Our data show that chronic cannabis use may be associated with an enhancement of oculomotor functional resiliency and suppression of the neuroinflammatory response following soccer heading. NPC has benefited from cannabis use in relation to faster recovery from 20 headings, while serum S100B level reflected the cannabis’s anti-inflammatory effects. A future study should incorporate a multimodal approach, such as diffusion and functional MRI, to explore what aspects of the brain are positively or negatively influenced by cannabis use.

## STAR★Methods

### Key resources table

**Table:** 

REAGENT or RESOURCE	SOURCE	IDENTIFIER
**Biological samples**		

Human venous serum	Peripheral human blood obtained by Kawata’s research team	N/A
Human urine samples	Human urine samples from participants	N/A
Human saliva samples	Human saliva samples from participants	N/A

**Critical commercial assays**		

SIMOA NF-LIGHT assay kits	Quanterix™	N/A
S100B ELISA	EMD Millipore Corporation	EZHS100B-33K
THC saliva test	NarcoCheck Inc.	CAT# NCE-S-THC-1
PreDosage THC urine test	NarcoCheck Inc.	CAT# DOA-M03-9B

**Software and algorithms**		

R version 3.4.1 with the package nlme	The R Project for Statistical Computing	N/A
Prism 9 (version 9.0.1)	GraphPad Software	N/A

**Other**		

Triaxial accelerometer - GForce Tracker	GForce Tracker Inc	N/A
Accommodative ruler for convergence	Gulden Ophthalmics	N/A
Trial registration	Clinicaltrials.gov	NCT04641832

### Resource availability

#### Lead contact

Further information and requests for resources should be directed to and will be fulfilled by the lead contact, Keisuke Kawata (kkawata@indiana.edu).

#### Materials availability

This study did not generate new unique reagents.

### Experimental model and study participant details

#### Trial design

This mechanistic clinical trial was a prospective evaluation of chronic cannabis effects on brain resiliency to acute subconcussive head impacts induced by 20 controlled soccer headings. The study consisted of two groups [cannabis and non-cannabis (control)], and participants were assessed for NPC, S100B, and NfL at 4-time points (pre-heading baseline, 2 h, 24 h, and 72 h post-heading). Time points were selected to gauge the acute and subacute phases of neural response after headings. Between pre- and 2 h post-heading, both groups performed 20 soccer headers (see Subconcussion Intervention section). Participants remained in the laboratory until the 2 h post-heading without engaging in strenuous cognitive or physical activities and returned to the laboratory approximately 24 and 72 h later. Participants were instructed to refrain from cannabis and alcohol use, aerobic exercise, and activities involving head impacts 72 h prior to the study to eliminate potential acute effects and throughout the study period. A salivary test was performed at each timepoint to ensure that the participants refrained from using cannabis during the study period. The Indiana University Institutional Review Board approved the study protocol, which was registered under ClinicalTrials.gov (ID: NCT04641832), and written informed consent was obtained from all participants.

#### Participants

From October 2020 through March 2021, 70 potential participants were screened, and 43 participants (cannabis n = 24 and non-cannabis n = 19) were included in the study (Figure 1). All potential participants filled out demographic and health questionnaires pertaining to his/her current health status, sports participation, and history of diagnosed concussion. General inclusion criteria across both groups consisted of being between the ages of 18 and 26 years old and having at least 5 years of soccer heading experience. Participants were asked to recall and self-report his/her history of diagnosed concussions. This retrospective recall method has the potential to be influenced by recall bias; however, it is a commonly used method and has been implemented in our previous studies.^28^^,^^30^^,^^52^ The sample size was determined based on our prior subconcussion studies^25^^,^^26^^,^^28^^,^^30^^,^^53^ and cannabis studies in humans,^8^^,^^9^^,^^40^ where 18 subjects per group were estimated to yield a statistical power of at least 0.80 with a significance level of α = 0.05.

### Method details

#### Validation of cannabis use

We have taken multiple steps to screen participants’ cannabis use history. First, chronic cannabis use was operationally defined as participants self-reporting the use of cannabis products on average once per week for the last 6 months, whereas the non-cannabis control group consists of those who had not used any cannabis products in the last 6 months. Participants completed a questionnaire gauging the types of cannabis product and frequency of cannabis use. Second, participants completed the Cannabis Use Disorder Identification Test – Revised (CUDIT-R), which is a brief, 8-item screening measure.^54^ It is a valid measure for the identification of likely cases of the Diagnostic and Statistical Manual of Mental Disorders (DSM-5) cannabis use disorder and is a screening tool to identify problematic cannabis use. Participants also completed the Alcohol Use Disorders Identification Test (AUDIT), which is a 10-item screening tool developed by the World Health Organization (WHO) to assess alcohol consumption, drinking behaviors, and alcohol-related problems.^55^^,^^56^ Third, a THC saliva test (NarcoCheck Inc.), with the lowest detection limit of 10 ng/mL up to 6 h after the last THC use, was used on days of testing to ensure that participants in both groups were free of acute THC effects. Fourth, urine samples were collected during the pre-intervention baseline testing. Using the PreDosage THC urine test (NarcoCheck Inc.), we verified whether the chronic cannabis users have in fact used cannabis in the past month, and vice versa, for the non-cannabis users. In summary, the chronic cannabis group consisted of participants with self-reported habitual cannabis use, a negative saliva result, and a positive urine result at any level. Conversely, the non-cannabis group consisted of participants with no cannabis use, negative saliva results, and a negative urine result.

#### Subconcussion Intervention

A standardized and reliable soccer heading protocol was used as a means to induce subconcussive head impacts.^23^,^26^^,^^30^ See Bevilacqua et al.^23^ for the video version of the soccer heading protocol. Briefly, a triaxial accelerometer (GForce Tracker Inc.) was secured inside a headband and positioned directly below the external occipital protuberance (inion) to measure linear and rotational head acceleration. A JUGS soccer machine (JPS Sports) was used to launch a size 5 soccer ball, with the ball traveling at a speed set at 25 mph (11.2 m/s). This ball speed is similar to when soccer players make a long throw-in from the sideline to midfield.^57^ Participants in both groups were situated 40 feet away from the machine to perform the headers and were allowed to adjust their position to complete a header with proper heading technique. Along with a tester’s demonstration, participants were instructed to head a ball in the air and aim for a tester standing approximately 16 feet in front of the participants. Participants performed 20 headers with a 30-s interval between each launch.

#### Near point of convergence

Our established protocol was used to measure participants’ NPC.^24^^,^^26^^,^^28^ No corrective spectacles were permitted; participants wore contact lenses. Participants were instructed to sit with their heads in a neutral anatomical position while using the accommodative ruler (Gulden Ophthalmics), and a target (14-point letter) was moved toward the eyes at a rate of 1–2 cm/s. The participant verbally stated when diplopia occurred or when the tester observed eye misalignment. Following the verbal cue, the tester stopped moving the target and the distance between the participant and the object was recorded in centimeters. The assessment was repeated twice, and the mean NPC value was used for analyses. We had 3 trained testers for this study (D.J.R., I.L.A., R.M.K.), whose mean intertester reliability was excellent (intraclass correlation coefficient, 0.93 [95% CI, 0.88–0.96]; p < 0.001)

#### Biomarker analysis

Serum S100B concentrations were measured using an enzyme-linked immunosorbent assay (ELISA) kit (EMD Millipore Corporation). The lower detection limit of the assay is 2.7 pg/mL using a 50 μL serum sample size, and the assay covers a concentration range of up to 2000 pg/mL, with an inter-assay variation of 1.9–4.4% and an intra-assay variation of 2.9–4.8%. Samples were loaded in duplicate into the ELISA plates according to manufacturer instructions. Fluorescence was measured by a microplate reader (BioTek EL800, Winooski, VT) and converted into pg/mL as per the standard curve concentrations. The S100B ELISA was performed by a member of the research team blinded to the group assignment information. Two participants showed serum S100B levels that were greater than 3 standard deviations from the mean at all time points; thus, their data were excluded from the final analyses.

Serum NF-L concentrations were measured using the Simoa SR-X Biomarker Detection System (Quanterix, Lexington, MA), a magnetic bead-based, digital ELISA that allows detection of proteins at subfemtomolar concentrations.^30^ The limit of detection (LOD) for the Simoa NF-L SR-X assay is 0.0552 pg/mL, and the lower limit of detection (LLOQ) is 0.316 pg/mL. The Simoa assay was performed by certified laboratory personnel blinded to group assignments. Serum samples from all subjects were assayed in duplicate, on the same plate. The average intra-assay coefficient of variation for the samples was 4.2%.

### Quantification and statistical analysis

#### Primary and secondary outcomes

The primary outcomes of the study were the group-by-time interactions of NPC, S100B, and NfL at 24-h post heading. The secondary outcomes included within-group and between-group analyses on NPC, S100B, and NfL at all time points, as well as testing for the diagnostic accuracy for outcomes that showed a significant group difference.

#### Statistical analysis

The demographic differences between the cannabis and non-cannabis groups were assessed by independent samples Student’s t-tests for continuous variables and chi-square tests for categorical variables. We examined the within-group and between-group pattern of NPC, S100B, and NfL, as reflected in primary and secondary outcomes, in response to 20 soccer headings using a series of mixed-effects regression models (MRM). The primary predictors (fixed effect) were group (cannabis vs. non-cannabis), time of measurement (pre, 2-h, 24-h, and 72-h post-heading), and the group by time interaction. The group differences were obtained by using a group-by-time interaction at each post-heading time point. We treated time as a categorical variable by providing dummy variables for each time and participants as a random effect to account for the repeated measures from the same participants. The model included potential covariates: sex, years of heading experience, number of concussions, CUDIT, and AUDIT. Since MRM was run per outcome measure (three outcomes in total), the significance level was corrected to p < 0.017 for the primary outcome analysis.

Outcome variables with a significant group-by-time interaction were further assessed for their classification ability at each post-heading time point using a receiver operating characteristic (ROC) analysis. The differences between post-heading time points and pre-heading baseline were used in the analysis, and an estimate of the area under the curve (AUC) with a 95% confidence interval (CI) was obtained. An AUC of 0.5 indicates no discrimination while an AUC of 1.0 indicates a perfect diagnostic utility. All analyses were conducted using the statistical software R version 3.4.1 with the package nlme and Prism 9 (version 9.0.1). The analysis was summarized by providing a contrast estimate with its 95% CI and a p value in the following format: [estimate (CI_low, CI_high); p value].

## Data Availability

• Data: All data reported in this paper will be shared by the lead contact upon request. • Code: This paper does not report original code. • Trial protocol registration in the ClinicalTrials.gov (ID: NCT04641832): https://clinicaltrials.gov/ct2/show/NCT04641832
